# Supplementary material for: A new basal ornithopod dinosaur from the Lower Cretaceous of China
Source: PeerJ. 2020 Sep 8;8:e9832. doi: 10.7717/peerj.9832 (PMC7485509; doi:10.7717/peerj.9832)
Supplement: Supplemental Information 1 — Descriptive list of characters used in the present analysis, grouped by major anatomical region, and based on the character list previously published by Dieudonné et al. (2016). Characters for which the definition and/or the coding was modified are marked with an asterisk. [file peerj-08-9832-s001.docx]

**Table S1. Character Description.**

Descriptive list of characters used in the present analysis, grouped by major anatomical region, and based on the character list previously published by Dieudonné et al. (2016). Characters for which the definition and/or the coding was modified are marked with an asterisk.

**Cranial skeleton**

1*. Skull, rostral**-**quadrate length relative to the body length: 10 % (0), 13 % or more (1) (Xu *et al.* 2006 #1; Ösi *et al.* 2012 #2).

2*. Skull, preorbital region, percentage out of the total skull length, from the premaxilla to the quadrate: equal or more than 40% (0), much less than 40% (1) (Xu *et al.* 2006 #21; Ösi *et al.* 2012 #1).

3*. Skull, position of maximum widening of the skull: beneath the jugal–postorbital bar (0), caudally, beneath the infratemporal fenestra (1) (Ösi *et al.* 2012 #37, Xu *et al.* 2006 #2).

4. Skull, widening of the skull across the jugals, chord from frontal orbital margin to extremity of jugal is more than minimum interorbital width: absent (0), present (1) (Ösi *et al.* 2012 #36).

5. Skull, cortical remodeling of surface of dermal bone: absent (0), present (1) (Ösi *et al.* 2012 #89).

6. Rostral bone (neomorphic bone anterior to premaxilla): absent (0), present (1) (Ösi *et al.* 2012 #3; Xu *et al.* 2006 #3).

7*. Rostral, shape of anterior face: round, convex (0) or sharply keeled (1) (Ösi *et al.* 2012 #4; Xu *et al.* 2006 #5).

8. Rostral bone, ventrolateral processes: rudimentary (0), well-developed (1) (Ösi *et al.* 2012 #5).

9*. Premaxilla, rostral and dorsal surfaces: lack rugosities (0), with rugosities (1) (Brown *et al.*, 2013 #136).

10*. Premaxilla, ventral inflection: absent, oral margin even with ventral margin of maxilla (0), present, oral margin projects farther ventrally than ventral margin of maxilla (1) (Xu *et al.* 2006 #37; McDonald *et al.* 2010 #30; Ösi *et al.* 2012 #9).

11. Premaxilla, denticles on oral margin: absent (0), present (1) (McDonald *et al.* 2010 #33).

12. Premaxilla, edentulous rostral region: absent, first premaxillary tooth is positioned adjacent to the symphysis (0), present, first premaxillary tooth is inset the width of one or more crowns (1) (Ösi *et al.* 2012 #6).

13*. Premaxilla, caudolateral process: does not contact lacrimal (0), contacts the lacrimal, excludes maxilla–nasal contact (1) (Xu *et al.* 2006 #34; Ösi *et al.* 2012 #7).

14. Premaxilla, ventral (or oral) margin: narial portion of the body of the premaxilla slopes steeply from the external naris to the oral margin (0), ventral premaxilla flares laterally to form a partial floor of the narial fossa (1) (Ösi *et al.* 2012 #8).

15. Premaxilla, premaxillary foramen: absent (0), present (1) (Ösi *et al.* 2012 #10).

16. Premaxilla, premaxillary palate: strongly arched, forming a deep, concave palate (0), horizontal or only gently arched (1) (Xu *et al.* 2006 #6; Ösi *et al.* 2012 #11).

17*. Premaxillae: unfused (0), fused (1) (Brown *et al.* 2013 #124).

18. Premaxilla, external naris size: small, entirely overlies the premaxilla (0), enlarged, extends posteriorly to overlie the maxilla (1) (Ösi *et al.* 2012 #18).

19*. Premaxillary internarial bar: present, reaches the nasal (0), incomplete or absent (1) (reformulated from Ösi *et al.* 2012 #12; Boyd, 2015 #11).

20. Premaxilla, position of the ventral margin of external nares: below the ventral margin of the orbits (0), above the ventral margin of the orbits (1) (Xu *et al.* 2006 #22; Ösi *et al.* 2012 #17).

21*. Premaxilla, narial fossa surrounding external nares on lateral surface of premaxilla, position of ventral margin of fossa relative to the ventral margin of the premaxilla: closely approaches the ventral margin of the premaxilla (0), separated by a broad flat margin from the ventral margin of the premaxilla (1) (Ösi *et al.* 2012 #16).

22*. Maximum length of external nares less than 15% basal skull length (0), maximum length of external nares greater than 15% basal skull length (1) (Boyd, 2015 #88).

23*. Premaxilla-maxilla contact, fossa-like depression positioned on the premaxilla–maxilla boundary: absent (0), present (1) (Ösi *et al.* 2012 #13).

24*. Premaxilla-maxilla diastema: weak to absent, maxillary teeth continue to anterior end of maxilla (0), present, substantial diastema of at least one crown length between maxillary and premaxillary teeth (1) (Ösi *et al.* 2012 #14).

25*. Premaxilla-vomer contact: present (0), absent, excluded by midline contact between maxillae (1) (Xu *et al.* 2006 #9; Ösi *et al.* 2012 #84).

26*. Maxilla, prominent anterolateral boss articulates with the medial premaxilla: absent (0), present (1) (Ösi *et al.* 2012 #24).

27*. Maxilla, buccal emargination: absent (0), present (1) (Ösi *et al.* 2012 #26).

28*. Maxilla, eminence on the rim of the buccal emargination of the maxilla near the junction with the jugal: absent (0), present (1) (Xu *et al.* 2006 #24; Ösi *et al.* 2012 #27).

29*. External antorbital fenestra, shape: triangular, the acute angle passes posteroventrally next to or below the orbit (0), oval or circular (1) (modified from Ösi *et al.* 2012 #22).

30*. External antorbital fenestra, exclusion of the jugal from the caudoventral margin by lacrimal–maxilla contact: absent (0), present (1) (Xu *et al.* 2006 #79; Ösi *et al.* 2012 #34).

31*. Internal antorbital fenestra, length relative to skull length: large, generally at least 15 % (0), very much reduced, less than 10% (1), or absent (2) (modified from Ösi *et al.* 2012 #20).

32*. Nasals, depression present along sutural line: absent (0), present (1) (reformulated from Xu *et al.* 2006 #82; Ösi *et al.* 2012 #19).

33*. Frontal, contacts orbit: along more than 25% of total frontal length (0), less than 25% (1), excluded from orbital margin (2) (modified from Brown *et al.* 2013 #24).

34*. Frontal, ratio of frontal length to nasal length: greater than 120% (0), between 120% and 60% (1) or less than 60% (2) (Brown *et al.* 2013 #25).

35. Frontals, short and broad (0), narrow and elongate (at least twice as long as wide) (1) (modified from Ösi *et al.* 2012 #64).

36*. Frontals arching over orbit from lateral view: present (0), absent, frontals dorsally flattened over orbit (1) (Boyd, 2015 #65).

37*. Lacrimal-jugal contact: jugal doesn’t, or barely touches lacrimal (0), jugal contacts lacrimal (1) (modified from Brown *et al.* 2013 #50).

38. Accessory ossification(s) in the orbit (palpebral/supraorbital): absent (0), present (1) (Xu *et al.* 2006 #68; Ösi *et al.* 2012 #29).

39. Palpebral/supraorbital: free, projects into orbit from contact with lacrimal/prefrontal (0), incorporated into orbital margin (1) (Ösi *et al.* 2012 #30).

40*. Palpebral, shape in dorsal view: rod-shaped (0), plate-like with wide base (1) (Ösi *et al.* 2012 #31).

41. Palpebral/supraorbital, number: one (0), two (1) or three (2) (Ösi *et al.* 2012 #32).

42*. Supraorbital(s) horizontal extension across the orbit (either fused or not to the orbital margin) : contact the postorbital posteriorly (0), does not contact the postorbital, but crosses at least half of the orbit (1), crosses less than half of the orbit (2) (modified from Boyd, 2015 #25).

43*. Lower margin of the orbit circular (0), lower margin of the orbit subrectangular (1) (Boyd, 2015 #95).

44. Postorbital-parietal contact: absent (0), very narrow (1), broad (2) (modified from Ösi *et al.* 2012 #51).

45*. Squamosal-Quadratojugal contact: present, between dorsal process of quadratojugal and descending process of the squamosal (0), absent (1) (Ösi *et al.* 2012 #52).

46*. Supratemporal fenestra length relative to the basal skull length (BSL): short, fenestrae are less than 25% BSL (0), elongated, more than 25% BSL (1) (Xu *et al.* 2006 #58; Ösi *et al.* 2012 #66).

47*. Parietosquamosal shelf with an overhang of the occipital region: absent (0), present (1) (reformulated from Ösi *et al.* 2012 #68).

48*. Postorbital-squamosal tubercle/node row: absent (0), present (1) (Ösi *et al.* 2012 #72).

49*. Postorbital-squamosal tubercle row, enlarged tubercle row on the posterior squamosal: absent (0), present (1) (Ösi *et al.* 2012 #73).

50*. Squamosal, morphology of postorbital process dorsal to *M. adductor mandibulae externus* origin site: gently convex (0), mediolaterally compressed and blade-like (1) (McDonald *et al.* 2010 #65).

51*. Jugal wing: degree of anteroposterior overlap of the quadrate shaft (not considering the pterygoid wing): greater than 50% quadrate length (0), less than 50% (1). (modified from Brown *et al.*, 2013 #1).

52*. Jugal, ventral extent of the jugal wing ends: at or near distal condyles of quadrate (0), above distal condyles (1), well above the distal condyles (2) (Brown *et al.* 2013 #9; Ösi *et al.* 2012 #54).

53*. Jugal, articulation with quadrate: jugal fails to articulate with quadrate (0), jugal articulates with quadrate (1) (Brown *et al.* 2013 #14).

54*. Maxillary process on the medial side of the jugal: straight to slightly arched medially (0), anteromedially projected and arched (1) (modified from Boyd, 2015 #39).

55. Jugal, ectopterygoid articular facet on medial view: consists of a deep groove (0), rounded scar (1) (Brown *et al.* 2013 #47).

56*. Jugal anterior ramus: dorsoventrally deeper than mediolaterally broad (0), broader than deep (1) (Boyd, 2015 #32).

57*. Jugal, morphology of portion of maxillary process that overlaps maxilla: tapers at anterior ends of maxillary and lacrimal contacts, with slightly convex ventral margin and slightly concave dorsal margin (0), subrectangular with parallel dorsal and ventral margins (1) (modified from: McDonald *et al.* 2010 #54; Ösi *et al.* 2012 #35).

58*. No boss present on lateral surface of the jugal (0), presence of a boss or horn on the lateral surface of the jugal (1) (modified from Boyd, 2015 #38).

59*. Jugal ornamentation: absent (0), present, nodular (1) (modified from Xu *et al.* 2006 #81; Ösi *et al.* 2012 #41).

60*. Jugal-postorbital bar, antero-posterior width relative to that of the infratemporal fenestra: less expanded (0), equally expanded (1), or anteroposteriorly broader than the infratemporal fenestra (2) (modified from Ösi *et al.* 2012 #42).

61*. Jugal-postorbital joint: elongate scarf joint (0), short butt joint (1) (Ösi *et al.* 2012 #43).

62*. Jugal, form of postorbital process: not expanded dorsally (0), dorsal portion of postorbital process expanded posteriorly (1) (Ösi *et al.* 2012 #44).

63*. Jugal, caudal ramus forking: absent (0); present, incision between processes narrow (1); present, incision between processes wide, forming an angle of more than 45° (2) (modified from Ösi *et al.* 2012 #46; Rozadilla *et al.* 2016, #46).

64*. Jugal, posterior ramus: forms anterior and ventral margin of infratemporal fenestra (0), forms part of posterior margin, expands towards squamosal (1) (Ösi *et al.* 2012 #47).

65*. Jugal–quadratojugal contact: overlapping (0), tongue-and-groove (1) (Ösi *et al.* 2012 #48).

66*. Jugal (or jugal–epijugal), ridge dividing the lateral surface of the jugal into two planes: absent (0), present (1) (Ösi *et al.* 2012 #38).

67*. Quadratojugal, shape: inverted L-shaped, with elongate anterior and ventral processes (0), subrectangular with long axis vertical, short, deep anterior process (1), horizontal T-shaped, with sharp angle between the anterior and dorsal processes (2) (modified from Ösi *et al.* 2012 #53).

68*. Quadratojugal, quadratojugal foramen: absent (0), present (1) (McDonald *et al.* 2010 #58).

69*. Paraquadratic foramen or notch, size: absent or small (0), large (1) (modified from Ösi *et al.* 2012 #60).

70*. Body of the quadrate leans posteriorly (0), body of quadrate oriented vertically (1), body of quadrate leans anteriorly (2) (Boyd, 2015 #47).

71*. Quadrate, prominent oval fossa on pterygoid ramus: absent (0), present (1) (Ösi *et al.* 2012 #57).

72*. Quadrate, mandibular articulation: quadrate condyles subequal in size (0), medial condyle is larger than lateral condyle (1), lateral condyle is larger than medial (2) (Ösi *et al.* 2012 #63).

73*. Laterosphenoid, socket for the head: occurs along frontal-postorbital suture (0), only in postorbital (1) (modified from Brown *et al.* 2013 #21).

74*. Post-temporal foramen position: at the boundary between the parietals/squamosals and the paroccipital process (0), entirely within the opisthotic (1), positioned entirely within the squamosal (2) (Boyd, 2015 #103; Rozadilla *et al.* 2016 #77).

75*. Opisthotic, presence of a ‘Y-shaped’ indentation on the dorsal edge for the passage of the post-temporal foramen: absent (0), present (1) (reformulated from Brown *et al.* 2013 #127).

76*. Prootic, position of the foramen for the trigeminal nerve (V): notches the anteroventral edge of the prootic (0), nearly or completely enclosed in prootic (1) (Brown *et al.*, 2013 #76).

77*. Prootic-basisphenoid plate: absent (0), present (1) (Ösi *et al.* 2012 #81).

78*. Supraoccipital, contribution to dorsal margin of the foramen magnum: forms entire dorsal margin of foramen magnum (0), exoccipital with medial process that restricts the contribution of the supraoccipital (1), both exoccipitals join medially and totally exclude the supraoccipital from the dorsal margin of the foramen magnum (2) (modified from Ösi *et al.* 2012 #78).

79*. Paroccipital processes (Exoccipital-Opisthotic complex): extend laterally and transit smoothly toward a slight dorsoventral expansion distally (0), distal end pendent, sharply deflects ventrally (1) (Xu *et al.* 2006 #94; McDonald *et al.* 2010 #72 ; Ösi *et al.* 2012 #75).

80*. Paroccipital processes, proportions: short and deep (height ≥ 1/2 length) (0), elongate and narrow (1) (Xu *et al.* 2006 #35; Ösi *et al.* 2012 #76).

81*. Basioccipital: foramen magnum occupies less than 50% of the width of occipital condyle (0), more than 50% of the width of the occipital condyle (1) (modified from Ösi *et al.* 2012 #79; Brown *et al.* 2013 #71).

82*. Basioccipital, orientation of occipital condyle: posteroventrally directed (0), posteriorly

directed (1) (McDonald *et al.* 2010 #74).

83*. Basioccipital, anteroposteriorly directed groove extending along ventral surface: absent (0), present (1) (McDonald *et al.* 2010 #75).

84*. Basioccipital, median ridge extending along ventral surface: absent (0), present (1) (reformulated from Xu *et al.* 2006 #95; McDonald *et al.* 2010 #76; Brown *et al.* 2013 #73).

85*. Basioccipital, basal tubera: extend much farther ventrally than the basisphenoid/parasphenoid plate (0), level (1) (reformulated from Brown *et al.* 2013 #74).

86*. Basioccipital, basal tubera: level with the base of the basioccipital condyle (0), form a massive buttress which extends much lower than the base of the basioccipital condyle (1) (reformulated from Ösi *et al.* 2012 #82).

87*. Angle between the base (i.e. occipital condyle, basisphenoid) and long axis of the braincase : less than 35 degrees (0), equal or more than 35 degrees (1) (reformulated from Boyd, 2015 #98).

88*. Basisphenoid, basipterygoid processes articular facet orientation: anteroventral and/or anterolateral (0), ventral (1), posteroventral (2) (Xu *et al.* 2006 #14; Ösi *et al.* 2012 #83).

89*. Basisphenoid, length relative to basioccipital length: longer or subequal (0), shorter than

basioccipital (1) (Ösi *et al.* 2012 #80).

90. Palatal keel, dorsoventrally deep (deeper than 50% of snout depth) median palatal keel formed of the vomers, pterygoids and palatines: absent (0), present (1) (Ösi *et al.* 2012 #85).

91*. Pterygoid-maxilla contact, at posterior end of tooth row: absent (0), present (1) (Ösi *et al.* 2012 #87).

92. Lower jaw, length of post-coronoid elements relative to the total length of the lower jaw: 35-40% (0), 25-35% (1) (modified from Brown *et al.* 2013 #62; Boyd, 2015 #83).

93*. Predentary: absent (0), present (1) (Ösi *et al.* 2012 #90).

94*. Predentary, oral margin size and position: short and the posterior extremity is posteriorly set, the predentary oppose only the first half of the premaxilla (0), short, the posterior border is anteriorly set, all but the posterodorsal corner of the predentary is positioned anterior to the last premaxillary tooth (1), roughly equal in length to the premaxilla, premaxillary teeth only oppose predentary all along (2), (modified from Ösi *et al.* 2012 #91).

95*. Predentary, shape: rounded (0), pointed (1) (Ösi *et al.* 2012 #92).

96. Predentary, grooves on either side of midline on anterior surface, extending ventrolaterally to dorsomedially: absent (0), present (1) (McDonald *et al.* 2010 #6).

97. Predentary, oral margin: relatively smooth (0), denticulate (1) (Ösi *et al.* 2012 #93).

98. Predentary, tip of in lateral view: does not project above the main body (0), strongly upturned relative to main body (1) (Ösi *et al.* 2012 #94).

99. Predentary, ventral process: single (0), bilobate (1) (Ösi *et al.* 2012 #95).

100. Predentary, ventral process: present, well-developed (0), very reduced or absent (1) (Ösi *et al.* 2012 #96).

101. Dentary, ratio of dentary height (just anterior to the rising coronoid process) divided by length of dentary: between 15-20% (0), 20-35% (1) (Brown *et al.* 2013 #63).

102*. Dentary, symphysis: V-shaped (0), spout shaped (1) (Ösi *et al.* 2012 #97).

103*. Dentary, position of the rostral tip: positioned high (0), mid height (1), near lower margin of dentary (2), below lower margin (3) (modified from: Brown *et al.* 2013 #51).

104. Dentary, morphology of ventral margin of rostral ramus leading to the predentary articulation: straight (0), inflected ventrally, such that it curves gently towards the predentary articulation and symphysis (1), curves dorsally towards symphysis in a continuous manner (2) (McDonald *et al.* 2010 #16).

105. Dentary, tooth row (and edentulous anterior portion) in lateral view: straight (0), anterior end downturned (1) (Ösi *et al.* 2012 #98).

106. Dentary, dorsal and ventral margins: converge anteriorly (0), subparallel (1), deepen anteriorly (2) (McDonald *et al.* 2010 #15; Ösi *et al.* 2012 #99).

107. Dentary, ventral flange: absent (0), present (1) (Ösi *et al.* 2012 #100).

108*. Dentary, orientation of tooth row relative to lateral surface of dentary: convergent anteriorly and posteriorly, bowed medially at mid-length, the tooth row ends anterior to, but along the same axis as that of the coronoid process (0), convergent anteriorly and divergent posteriorly so that the tooth row ends medial to the coronoid process longitudinal axis, the last tooth is still anterior to the coronoid process (1), the last dentary tooth is medial to the coronoid process (2) (modified from Xu *et al.* 2006 #17; McDonald *et al.* 2010 #12 and Ösi *et al.* 2012 #103).

109*. Dentary, coronoid process: absent or weak, posterodorsally oblique, depth of mandible at coronoid is less than 150% depth of mandible beneath tooth row (0), well-developed, distinctly elevated, depth of mandible at coronoid is more than 150% depth of mandible beneath tooth row (1) (Rozadilla *et al.* 2016 #101).

110. Dentary, number of dentary teeth: 10 or fewer (0), 11–13 (1), 14–17 (2), 18 or more (3) (Ösi *et al.* 2012 #228) (ordered character).

111. Dentary, the posterolateral surface bears a profound circular depression: absent (0), present (1) (Ösi *et al.* 2012 #230).

112. External mandibular fenestra, situated on dentary-surangular-angular boundary: present (0), absent (1) (Ösi *et al.* 2012 #104).

113. Retroarticular process: long, subequal to or exceeding the length of the glenoid (0), rudimentary or absent (1) (reformulated from Xu *et al.* 2006 #29; Ösi *et al.* 2012 #107).

114. Dentary-angular, node-like ornamentation: absent (0), present (1) (Ösi *et al.* 2012 #108).

115. Jaw, level of jaw joint: weakly to very depressed ventrally by respect with the dentary tooth row (0), level with or dorsal to the dentary tooth row (1) (modified from Ösi *et al.* 2012 #109).

116. Premaxillary teeth: present (0), absent, premaxilla edentulous (1) (Ösi *et al.* 2012 #111).

117. Premaxillary teeth, crown expanded above root: absent (0), present (1) (reformulated fom Xu *et al.* 2006 #66; Ösi *et al.* 2012 #113).

118*. Premaxillary teeth size: all premaxillary teeth are subequal in size and not significantly broader than the succeeding maxillary teeth (0), posterior premaxillary teeth larger in size and height posteriorly (1) (Xu *et al.* 2006 #73; Ösi *et al.* 2012 #114).

119*. Teeth, crown is mesiodistally expanded above root in cheek teeth: absent (0), present (1) (Ösi *et al.* 2012 #129).

120*. Teeth, close-packing and quicker replacement eliminating spaces between alveolar border and crowns of adjacent functional teeth: absent (0), present (1) (Ösi *et al.* 2012 #131).

121. Teeth, wear facets on teeth: absent or sporadically developed (0), systematic development of wear facets along the entire tooth row (1) (Ösi *et al.* 2012 #222).

122. Maxillary/dentary teeth, position of maximum apicobasal crown height in tooth rows: anterior portion of tooth row (0), central portion of tooth rows (1), posterior portion of tooth rows (2) (Ösi *et al.* 2012 #130).

123. Maxillary/dentary teeth, marginal ornamentations: fine serrations set at right angles to the margin of the tooth (0), coarse serrations (denticles) angle upwards at 45 degrees from the margin of the tooth (1) (Ösi *et al.* 2012 #116).

124*. Maxillary/dentary teeth: enamel symmetrical (0), asymmetrical (1) (Ösi *et al.* 2012 #117).

125*. Maxillary/dentary teeth, apicobasally extending primary and secondary ridges: absent (0), present (1) (reformulated from Ösi *et al.* 2012 #118).

126*. Maxillary/dentary teeth, at least moderately developed labiolingual expansion of crown (‘cingulum’): absent (0), present (1) (reformulated from Ösi *et al.* 2012 #123).

127. Maxillary teeth, overlapping of adjacent crowns in maxillary teeth: non-packed maxillary teeth (0), lack of space between adjacent maxillary teeth up through the occlusional margin (1) (Xu *et al.* 2006 #103; McDonald *et al.* 2010 #88; Ösi *et al.* 2012 #128; Brown *et al.*, 2013 #31).

128*. Maxillary/dentary alveolar foramina (‘special foramina’) medial to tooth rows: present (0), absent (1) (Ösi *et al.* 2012 #126).

129. Maxillary teeth, crown shape: lingually concave (0), lingually convex (1) (Brown *et al.*, 2013 #37).

130*. Maxillary teeth, crowns shape: relatively low spade-like, rectangular, or triangular (0), high diamond-shaped maxillary tooth crowns (1) (Brown *et al.* 2013 #41).

131*. Maxillary teeth, apical ridge position, centrally placed (0), posteriorly set (1) (Brown *et al.* 2013 #38).

132*. Maxillary teeth, relative prominence of the primary ridge on labial surface of crown: no primary ridge (0), outstanding by comparing to other secondary ridges (1), completely undistinguishable from other secondary ridges in at least some maxillary teeth (2) (modified from McDonald *et al.* 2010 #92, Ösi *et al.* 2012 #120).

133. Maxillary teeth, root shape: straight (0), curved (1) (Brown *et al.* 2013 #33; Boyd, 2015 #119).

134. Dentary dentition, heterodonty: no substantial heterodonty is present in dentary dentition (0), single, enlarged, caniform anterior dentary tooth, crown is not mesiodistally expanded above root (1), anterior dentary teeth are strongly recurved and caniform, but have crowns expanded mesiodistally above their roots and are not enlarged relative to other dentary teeth (2) (Ösi *et al.* 2012 #124).

135. Dentary teeth, peg-like tooth located anteriorly within dentary lacks denticles, strongly reduced in size: absent (0), present (1) (Ösi *et al.* 2012 #125).

136. Dentary teeth, intercrown spaces: present (0), absent (1) (McDonald *et al.* 2010 #80).

137. Dentary teeth, crown shape: rectangular, triangular, or leaf-shaped (0), lozenge-shaped (1) (Brown *et al.* 2013 #60).

138. Dentary teeth, apical ridge position: anteriorly or centrally positioned (0), posteriorly positioned (1) (Brown *et al.* 2013 #52).

139. Dentary teeth, number of ridges on crown: fewer than 10 (0), more than 10, and often more than 17 (1) (Ösi *et al.* 2012 #229).

140. Dentary teeth, prominent primary ridge on lingual side: absent (0), present (1) (Ösi *et al.* 2012 #121).

141*. Dentary teeth, number and morphology of secondary and accessory ridges on lingual surface of the crown: no secondary ridges, separate faint accessory ridges arising from marginal denticles on both sides of the primary ridge (0), multiple parallel and evenly-spaced secondary ridges on either side such that entire lingual surface is corrugated (1), a few parallel and well defined secondary ridges with multiple faint accessory ridges arising from marginal denticles (2) (modified from McDonald *et al.* 2010 #87).

142*. Ridges present on both sides of dentary crowns (0), ridges limited to one side of dentary crowns (1) (Boyd, 2015 #124).

143. Dentary teeth, root shape in cross-section: round (0), oval (1), squared (2) (Dieudonné *et al.* 2016a #161, previously modified from Brown *et al.* 2013 #58).

144. Dentary tooth roots straight in anterior or posterior view (0), dentary tooth roots curved in anterior or posterior view (1) (Boyd, 2015 #135).

**Axial skeleton:**

145. Postaxial cervical vertebrae, epipophyses on anterior cervicals: present (0), absent (1) (Ösi *et al.* 2012 #133, Cambiaso, 2007 #3).

146. Cervical vertebrae (4-9), form of central surfaces: amphicoelous (0), at least slightly opisthocoelous (1) (Ösi *et al.* 2012 #134).

147*. Ventral surface of the cervical vertebrae : rounded (0), presence of a broad, flattened keel on the ventral surface of the cervical vertebrae (1), presence of a sharp ventral keel on the ventral surface of the cervical vertebrae (2) (Boyd, 2015 #143).

148*. Posterior cervical centra less than 1.5 times longer than tall (0), length of posterior cervical centra equal or greater than 1.5 times longer than tall (1) (modified from Boyd, 2015 #144).

149*. Cervical vertebrae, number: 8 or less (0), 9 (1), 10 or more (2) (Ösi *et al.* 2012 #135).

150*. Dorsal vertebrae, number: 12–13 (0), more than 14 (1) (modified from Ösi *et al.* 2012 #137).

151. Dorsal vertebrae, neural spine: anteriorly positioned or centered over the dorsal centrum (0), posteriorly positioned (1) (Brown *et al.* 2013 #78).

152*. Sacrum composed of three or fewer fused vertebral centra (0), sacrum composed of between four and five fused vertebral centra (1), sacrum composed of six fused vertebral centra (2), sacrum composed of seven or more fused vertebral centra (3) (Boyd, 2015 #148).

153. Sacral vertebrae, neural spines height: less than 2 times the height of the centrum (0), neural spines between 2 and 2,5 times the height of the centrum (1), greater than 2,5 times (2) (Brown *et al.* 2013 #82).

154. Sacrum, accessory articulation with pubis: pubis does not articulate with the sacrum (0), pubis supported by sacral rib (1), pubis supported by sacral centrum (2) (Ösi *et al.* 2012 #139; Brown *et al.* 2013 #84).

155. Ischiac peduncle of the ilium is not supported by a sacral rib (0), ischiac peduncle of the ilium supported by a sacral rib (1) (Boyd, 2015 #190).

156. Caudal vertebrae, neural spine position: caudal neural spines positioned over centrum (0), extend beyond own centrum (1) (Brown *et al.* 2013 #88).

157*. Anterior caudal vertebrae, neural spines: height the same or up to 50% taller than the centrum (0), more than 50% taller than the centrum (1) (Ösi *et al.* 2012 #142).

158. Dorsal ribs, transition between a near vertical orientation of the *tuberculum* and *capitulum* to a horizontal orientation: occurs within ribs 2-4 (0), 5-6 (1), 6-8 (2) (Brown *et al.* 2013 #79).

159. Anterior dorsal ribs, distal portions of the shaft in cross-section: circular or oval (0), highly laterally compressed with concave lateral and rugose posterior surfaces (1) (Brown *et al.* 2013 #135).

160. Partial ossification of the sternal segments of the cranial dorsal ribs absent (0), present (1) (Ösi *et al.* 2012 #145; Boyd, 2015 #157).

161*. Ossified epaxial tendons along dorsal and sacral vertebrae: absent (0), present (1) (new character).

162*. Caudal ribs, location: borne on centrum (0), on neurocentral suture (1), on neural arch (2) (Brown *et al.* 2013 #85).

163. Caudal ribs, longest rib position: the first caudal vertebra bears longest rib (0), longest rib distal to the first (1) (Brown *et al.* 2013 #87).

164*. Chevrons, shape: rod-shaped, often with slight distal expansion (0), strongly asymmetrically expanded distally, width greater than length in mid caudals (1) (Ösi *et al.* 2012 #144).

165*. Ossified epaxial tendons along caudal vertebrae: absent (0), present (1) (modified from Ösi *et al.* 2012 #216; Ösi *et al.* 2012 #217; Brown *et al.* 2013 #86).

166. Ossified epaxial tendons, arrangement: longitudinally arranged (0), Basket-like arrangement of fusiform tendons in caudal region (1), double-layered lattice (2) (modified from Ösi *et al.* 2012 #218).

**Appendicular skeleton:**

167. Ossified clavicles: absent (0), present (1) (Ösi *et al.* 2012 #147).

168*. Scapula-Humerus, proportions: scapula longer or subequal to the humerus (0), humerus substantially longer than the scapula (1) (Ösi *et al.* 2012 #149).

169*. Scapula, blade-shape: strongly expanded distally (0), weakly expanded, near parallel-sided (1) (Ösi *et al.* 2012 #152).

170. Scapula, scapular blade length relative to minimum width: relatively short and broad, length is 5-8 times minimum width (0), elongate and strap-like, length is at least 9 times the minimum width (1) (Ösi *et al.* 2012 #150).

171*. Scapula, acromion shape: weakly developed or absent (0), well-expanded anteriorly, spine-like (1) (reformulated from Ösi *et al.* 2012 #151).

172*. Scapula, angle formed by the proximal and distal edges of the cranioventral buttress: acute, less than 75° (0), more than 75° (1) (modified from Xu *et al.* 2006 #20; Dieudonné *et al.* 2016a #191).

173*. Scapula and coracoid, undistinghuishably fused into a scapulocoracoid: absent (0), present (1) (new character).

174. Coracoid, width divided by length: less than 60% (0), between 70% and 100% (1) or greater than 100% (2) (Brown *et al.* 2013 #90).

175. Coracoid, coracoid foramen position: enclosed within coracoid (0), open along coracoid-scapula suture (1) (Brown *et al.* 2013 #91).

176. Coracoid, development of the sternal process: short and broad (0), extremely elongated and narrow (ratio greater than 0.80) (1) (Ösi *et al.* 2012 #231).

177. Sternal plates, shape: absent (0), kidney-shaped (1), shafted or hatchet-shaped (rod-like posterolateral process, expanded anterior end) (2) (Ösi *et al.* 2012 #148).

178. Humerus, length relative to femoral length: more than 60% (0), less than 60% (1) (Ösi *et al.* 2012 #153).

179. Humerus, appearance of the anterior surface in proximal view: a varyingly developed flexor bicipital sulcus is visible (0), the anterior surface is completely straight to smoothly convex, no bicipital sulcus visible (1) (Dieudonné *et al.* 2016a #197).

180. Humerus, proximal end in anterior/posterior view, lateral border between head and deltopectoral crest: straight or gently convex (0), concave (1) (Ösi *et al.* 2012 #232).

181. Humerus, head separated from prominent medial tubercle on proximal surface by a groove: absent (0), present (1) (Ösi *et al.* 2012 #223).

182*. Humerus, deltopectoral crest development: well-developed or at most a thickening that projects anteriorly as a distinct flange (0), almost imperceptible (1) (Ösi *et al.* 2012 #154).

183. Humerus, deltopectoral crest shape: distal margin rounded and merges gradually with the lateral margin of the humeral shaft (0), distal margin angular and merges abruptly with the lateral margin of the humeral shaft (1) (McDonald *et al.* 2010 #103).

184. Humerus, shaft form in cranial and caudal views: relatively straight (0), strongly bowed laterally along length (1) (Ösi *et al.* 2012 #155).

185. Ulna, olecranon process: low (0), moderately developed (1), relatively high (2) (Brown *et al.* 2013 #93).

186. Ulna, distal end: directed ventrally in medial or lateral view (0), curves gently posteriorly in medial or lateral view (1) (Ösi *et al.* 2012 #233).

187. Carpus, fusion: unfused (0), fused (1) (Brown *et al.* 2013 #97).

188. Metacarpals, block-like proximal ends: absent (0), present (1) (Ösi *et al.* 2012 #157).

189. Metacarpals I and V: substantially shorter in length than metacarpal III (0), subequal in length to metacarpal III (1) (Ösi *et al.* 2012 #158).

190. Metacarpal I greater than 50% the length of metacarpal II (0), metacarpal I less than 50% the length of metacarpal II (1) (Boyd, 2015 #174).

191. Metacarpal/manual phalanges, extensor pits on the dorsal surface of the distal end: absent or poorly developed (0), deep, well-developed (1) (Ösi *et al.* 2012 #162).

192. Manual phalanx, the longest length relative to humerus length: less than 10% (0), more than 15% (1) (Ösi *et al.* 2012 #156).

193. Penultimate phalanx of fingers II and III: shorter than first phalanx (0), longer than the first phalanx (1) (Ösi *et al.* 2012 #159).

194. Manual digits II–IV: First phalanx relatively short compared to second phalanx (0), first phalanx more than twice the length of the second phalanx (1) (Ösi *et al.* 2012 #161).

195. Manual digit III, number of phalanges: 4 (0), 3 or fewer (1) (Ösi *et al.* 2012 #160).

196. Manual unguals, strongly recurved with prominent flexor tubercle: absent (0), present (1) (Ösi *et al.* 2012 #163).

197. Ilium, preacetabular process shape and length: short, tab-shaped, distal end is posterior to pubic peduncle (0), elongate, strap-shaped, distal end is anterior to pubic peduncle (1) (Ösi *et al.* 2012 #165).

198*. Ilium, preacetabular process length relative to the ilium length: less than 50% (0), more than 50% (1) (Ösi *et al.* 2012 #166).

199. Ilium, lateral deflection of the preacetabular process: 10°-20° degrees from midline (0), more than 30° (1) (Ösi *et al.* 2012 #167).

200. Ilium, dorsal margin of preacetabular process and dorsal margin above acetabulum: narrow, not transversely expanded (0), dorsal margin is transversely expanded to form a narrow shelf (1) (Ösi *et al.* 2012 #168).

201. Ilium, preacetabular process expands mediolaterally towards its distal end in dorsal view: absent (0), present (1) (Ösi *et al.* 2012 #169).202*.

202*. Ilium, medioventral acetabular flange, partially closing the acetabulum: present (0), absent (1) (Ösi *et al.* 2012 #175).

203*. Ilium, supra-acetabular ‘crest’ or ‘flange’: present (0), absent (1) (Ösi *et al.* 2012 #176).

204*. Ilium, postacetabular process: posteriorly directed (0), curves posterodorsally with both its dorsal and ventral margins (1) (McDonald *et al.* 2010 #114; Ösi *et al.* 2012 #170).

205*. Ilium, morphology of dorsal margin of postacetabular process dorsal to ischial peduncle: thin all along or smoothly enlarged dorsal to ischial peduncle, no modification of dorsal margin (0), well thickened dorsal margin compared to dorsal margin above pubic peduncle (1) or thickened and laterally-bulging everted rim along dorsal margin (2) (McDonald *et al.* 2010 #112; Dieudonné *et al.* 2016a #222)).

206*. Ilium, dorsal surface of postacetabular process until the origin of *M. iliocaudalis*: smoothly convex with a posterior break in slope (0), concave, the postacetabular blade looks strongly quadrangular-shaped (1), tapers with no break in slope for the attachment of *M. iliocaudalis* (2) (modified from McDonald *et al.* 2010 #113; Dieudonné *et al.* 2016 #223).

207. Ilium, brevis shelf and fossa: faces ventrolaterally and shelf is near vertical and creates a deep postacetabular portion anteriorly (0), fossa faces ventrally and is hardly visible from a lateral view (1), no brevis shelf (2) (reformulated from Ösi *et al.* 2012 #173; Dieudonné *et al.* 2016 #224).

208*. Ilium, length of the postacetabular process relative to the total length of the ilium: 20% or less (0), 25-35% (1), more than 35% (2) (Ösi *et al.* 2012 #174).

209. Ilium, pubic peduncle: large, elongate, robust (0), reduced in size, shorter in length than ischial peduncle (1) (Ösi *et al.* 2012 #178).

210. Ilium, ischiac peduncle: projects ventrally (0), broadly swollen, projects ventrolaterally (1) (Ösi *et al.* 2012 #177).

211*. Ilium, acetabulum: normal to high (0), low (1) (reformulated after Boyd, 2015 #182).

212. Pubis, massive and dorsolaterally rotated body obscuring the obturator foramen in lateral view: absent (0), present (1) (Ösi *et al.* 2012 #191).

213. Pubis, orientation: anteroventral (0), rotated posteroventrally to lie alongside the ischium (opisthopubic) (1) (Ösi *et al.* 2012 #186).

214. Pubis, prepubic process: absent (0), present (1) (Ösi *et al.* 2012 #192).

215. Pubis, prepubic process shape: compressed mediolaterally, dorsoventral height exceeds mediolateral width (0), rod-like, mediolateral width exceeds dorsoventral height (1), dorsoventrally compressed (2), twisted along length – dorsoventrally compressed at its base and transversely compressed distally (3) (Ösi *et al.* 2012 #193).

216. Pubis, prepubic process length: stub-like and poorly developed, extends only a short distance anterior to the pubic peduncle of the ilium (0), elongated into distinct anterior process (1) (Xu *et al.* 2006 #106; Ösi *et al.* 2012 #194).

217. Pubis, prepubic process extending beyond distal end of preacetabular process of ilium: absent (0), present (1) (Ösi *et al.* 2012 #195).

218. Angle between prepubic process and pubic shaft greater than 150 degrees (0), angle less than 130 degrees (1) (Boyd, 2015 #196).

219. Pubis, pubic symphysis extending: elongate (0), restricted to distal end of pubic blade, or absent (1) (Ösi *et al.* 2012 #196).

220. Pubis, shape of the postpubis shaft in cross-section: blade-shaped (0), rod-shaped (1) (Ösi *et al.* 2012 #187).

221. Pubis, length of postpubis shaft relative to ischium length: approximately equal (0), extends for around half the length (1), very short to absent (2) (McDonald *et al.* 2010 #117 ; Ösi *et al.* 2012 #188, #189).

222*. Ischium, pubic peduncle shape: transversely compressed (0), dorsoventrally compressed (1) (Ösi *et al.* 2012 #179).

223. Pubic peduncle of ischium larger than iliac peduncle (0), peduncles subequal or iliac peduncle larger than pubic peduncle (1) (Boyd, 2015 #200).

224*. Ischium, tab-shaped obturator process: absent, lacks an obturator process (0), present and placed 60% down the shaft of ischium (1), placed within the first proximal half of the shaft (2) (modified from: Xu *et al.* 2006 #44; Ösi *et al.* 2012 #184; Brown *et al.* 2013 #102).

225. Ischium, shaft in cross-section: dorsoventrally compressed (0), subcircular and bar-like (1) (Ösi *et al.* 2012 #181).

226. Ischium, symphysis length: median symphysis with the opposing blade along at least 50% of its length (0), symphysis only presents distally (1) (Ösi *et al.* 2012 #185).

227. Femur, shape in medial/lateral view: bowed anteriorly along length (0), straight (1) (McDonald *et al.* 2010 #121; Ösi *et al.* 2012 #197).

228. Femur, femoral head: confluent with greater trochanter, *fossa trochanteris* is smooth and groove-like (0), *fossa trochanteris* is modified into distinct constriction separating head and greater trochanter (1) (Ösi *et al.* 2012 #198).

229*. Anterior trochanter, level with respect to the greater trochanter: well below (0), slightly below or level (1) higher (2) (modified from: Ösi *et al.* 2012 #200; Boyd, 2015 #215).

230*. Lesser trochanter of femur positioned anterior and medial to greater trochanter (0), lesser trochanter positioned anterior and somewhat lateral to greater trochanter (1) (Boyd, 2015 #216).

231*. Lateral surface of the greater trochanter of femur convex (0), lateral surface of the greater trochanter flattened (1) (Boyd, 2015 #213).

232. Femur, fourth trochanter shape: low eminence or absent (0), straight ridge (1), pendent (2) (Ösi *et al.* 2012 #201).

233. Femur, fourth trochanter position: located entirely on proximal half of femur (0) or positioned at mid-length, or distal to mid-length (1) (Ösi *et al.* 2012 #202).

234. Femur, pendent fourth trochanter, rod-like with subparallel anterior and posterior surfaces: absent (0), present (1) (Ösi *et al.* 2012 #224).

235. Femur, location of insertion scar of *M. caudifemoralis longus*: extends from fourth trochanter onto medial surface of femoral shaft (0), widely separated from fourth trochanter, restricted to medial surface of femoral shaft (1) (McDonald *et al.* 2010 #125).

236*. Femur, anterior (extensor) intercondylar groove: absent (0), shallow open through with sides that diverge from each other cranially (1), deep open through with parallel sides (2) (McDonald *et al.* 2010 #127; Ösi *et al.* 2012 #203).

237*. Femur, posterior (flexor) intercondylar groove: fully open (0), medial condyle inflated laterally, partially covers opening of flexor groove (1) (Ösi *et al.* 2012 #204).

238. Femur, posterolateral condyle position and size in ventral view: positioned relatively laterally and slightly narrower in width than the medial condyle (0), strongly inset medially, reduced in width relative to medial condyle (1) Ösi *et al.* 2012 #205).

239. Femur, cranial expansion of medial condyle: equal to, or less than lateral condyle (0), protrudes cranially to lateral condyle, and continues onto the cranial surface as a diaphyseal ridge to cranial trochanter (1) (Herne, 2014 #233; Dieudonné *et al.* 2016a #261).

240. Tibia, distal shape: subquadrate, posterolateral process not substantially developed (0), elongate posterolateral process, backing fibula (1) (Ösi *et al.* 2012 #206).

241. Tibia, maximum expansion of distal end relative to proximal: distal end is considerably less expanded than proximal (0), maximum expansion of distal end is subequal to that of proximal end (1) (Ösi *et al.* 2012 #227).

242. Fibula, shaft in cross-section: elliptical or round (0), D-shaped (1) (Brown *et al.* 2013 #115).

243*. Fibula, distal end is strongly reduced and splint-like: absent (0), present (1) (Ösi *et al.* 2012 #225).

244*. Astragalus, anterior process: moderate to high, from tooth-like to wide anteriorly (0), low to absent (1) (reformulated from Brown *et al.* 2013 #118).

245*. Astragalus, posterior side size: low (0), high (1) (Brown *et al.*, 2013 #117).

246*. Astragalus, fibular facet on the lateral margin of the proximal surface: large (0), reduced to small articulation (1) (Ösi *et al.* 2012 #207).

247*. Calcaneum, tibial articular surface from a lateral view: facet for tibia absent (0), facet for tibia present and subequal in length to that for the fibula (1), facet for tibia longer than the facet for the fibula, and the posteroventral part of the calcaneum is elongated into a distinct caudal process (2) (Ösi *et al.* 2012 #208, Rozadilla *et al.* 2016 #236).

248*. Calcaneum, posterior angle between the edge separating the tibial and fibular articular facets, and the lateral border of the calcaneum: greater than 110 degrees (0), less than 110 degrees (1) (modified from Brown *et al.* 2013 #119).

249*. Medial distal tarsal, shape: blocky in dorsal view (0), thin and rectangular (1), round (2) (Brown *et al.*, 2013 #120).

250*. Medial distal tarsal: articulates distally with metatarsal III only (0), articulates distally with metatarsals II and III (1) (Ösi *et al.* 2012 #209).

251*. Lateral distal tarsal, shape in dorsoventral view: square (0), kidney-shaped (1) (Brown *et al.* 2013 #122).

252. Metatarsal II/metatarsal III, morphology of the contact in proximal view: continuous and rather flat (0), metatarsal II overlaps a proximal outgrowth on the ventro-medial side of the metatarsal III (1) (Dieudonné *et al.* 2016a #277).

253*. Metatarsals III and IV, deep caudolateral notch on MT III for the reception of a prominent process of MT IV: absent (0); present (1) (McDonald *et al.* 2010 #134; Dieudonné *et al.* 2016a #278).

254. Metatarsal III and IV, proximal contact: tightly adpressed, no concavity is observed posteriorly between them (0), conspicuous concavity to either, or both, the posterolateral side of metatarsal III and the posteromedial side of metatarsal IV which can eventually host the fifth metatarsal (1) (Dieudonné *et al.* 2016 #279).

255. Metatarsal V, length relative to that of metatarsal III: more than 50% (0), less than 25% (1) (Ösi *et al.* 2012 #213).

256. Metatarsal V: bears digits (0), lacks digits (1) (Ösi *et al.* 2012 #214).

257. Pes, number of functional digits (i.e., bear phalanges): four (0), three (1) (Brown *et al.*, 2013 #123).

258*. Pedal digit I, configuration: robust and well-developed metatarsal I, distal end of phalanx I-1 projecting beyond the distal end of metatarsal II (0), reduced metatarsal I, proximally splint like, end of phalanx I-1 not extending beyond the end of metatarsal II (1), reduced metatarsal I in a vestigal splint or absent, not bearing digits (2) (Ösi *et al.* 2012 #211).

259. Pedal unguals, shape: tapering, narrow pointed, claw-like (0), wide, blunt, hoof-like (1) (Xu *et al.* 2006 #105; Ösi *et al.* 2012 #215).

**Dermal skeleton:**

260. Mandibular osteoderm: absent (0), present (1) (Ösi *et al.* 2012 #110).

261. Dermal osteoderms, parasagittal row on the dorsum of the body: absent (0), present (1) (Ösi *et al.* 2012 #219).

262. Dermal osteoderms, lateral row of keeled dermal osteoderms on the dorsum of the body: absent (0), present (1) (Ösi *et al.* 2012 #220).

263. Dermal osteoderms, U-shaped cervical/pectoral collars composed of contiguous keeled osteoderms: absent (0), present (1) (Ösi *et al.* 2012 #221).
