# Supplementary material for: A new basal ornithopod dinosaur from the Lower Cretaceous of China
Source: PeerJ. 2020 Sep 8;8:e9832. doi: 10.7717/peerj.9832 (PMC7485509; doi:10.7717/peerj.9832)
Supplement: Supplemental Information 3 [file peerj-08-9832-s003.docx]

xread

263 61

Herrerasaurus_ischigualastensis 000000--0000000000011000?0001001020?10----010000-0000??00000001000210201?0??0000000??0?0?0000-------00-?000?020000000000000001011---?0002-?0????00?1210000000??00?000???11011?0??00?10?0200100111001000000001001000000???-000000100000010?000000000?000??00000000000000

Abrictosaurus_consors 01?000--?1?1001??0000?01??10001??????1000?????????02???0??????????0?????????????????????????12??00?1?0??0?0?120000?00010111?000??????01???0????????????1????????1??????00?0??????0???0?0???10?1??0??10?001?0?01100????????????????0?11?201?0???1??1???????????11?100000

Agilisaurus_louderbacki 001000--0001001100000000??100011010001011001?000-?0?0???00000000001000110???0010?????0??0001111?0000?1?0000?13010000111011100010?010?200??000???10??11?1?01?0??011?0000100010?00?1000000?0???0??????100000000001100011010?11000200000??2000000011?0??11??0????110100000

Anabisetia_saldiviai ??????????????????????????1??????????????????????????????????????????????????????0011???????????????????0?011?????????1???1?111?1001????00??1???0?21?????????????2??10?000010110?000010010000?000??011100110001111001101101100120101110200?11111???1112?2??11?110100000

Ankylosauria 00?010--???10011?0?00?000010??10??0??11?20?1??00-??????????11000001?0?01????0000?????0?1110?100?00?1?1??100?0??1?0001010[0 1]11001?0?????00????0????10??0???????0??????0100010??????10??00?0???01?0?001011?10000??00001111000?1111-00110???01???00?11?0??1????????11?211111

Archaeoceratops_oshimai 10110111?0?10010?0001000??110010?20?110101???11101010??00?120100011000?2?????00110???????0??121?0110?10?00021??111001010111??0?0?????00??????????????0?????????????0?0??????????????????????????????1000011???1211?011210?111??001?11??????????1???00110?1?000110100000

Aurorella 000100--100?10??10000011?01001110110110100011000-0010???000010000010?0?????????????????????1111???????00000?1?01?0001110?1??101??0-0?0??0???????1??00103???01?0?110?00?100111100?0???0?010??????????100001100002000?????????????000?001????1??01?00?????????????0000000

Camptosaurus_dispar 000000--0111110101?0010?001000101101110001011000-002001?000000010000??0001000110001110120001120110101121010112010011??1111111110111110011101212111?1111?201110001110?20000000110?000000010100100011010?00110?0111110110111111112110?110210021101100111?020????111100000

Changchunsaurus_parvus 00?000--1001?011100000110?100?11????110?0?0?1??0-0010??00110??100010?000?????0???00??0?00001121101101110020112010000101011100110?0?0?00100000???101011????????001????0??00????001?00001???????????????????????1??1?????????????????111???0?0???1??0???1?????????0100000

Chaoyangsaurus_youngi ?1110100?0?1?0?0?0?11?00??11??1???????????????????0101??011???1000100??2?????????????????0??121?01?0?1??00001??111001010111100?0?????00????0?????0????????????????????????01???????????????????????????????????????????????????????????????????????????????????????0???

Dryosaurus_altus 000000--010111010100010?001010100100110000011000-011100000000001001011??00010110100000110000120?10?01110010112010001??1111111110111110011101211111111102101111001200?0000001001010000000001??0??????100001100111110011011111010211011102001211011?011121111100111200000

Dysalotosaurus_lettowvorbecki ?0?000--0?0111010100010?001001100100110001011000-012100000000001001111?2?001011010?010110000120110101110010111010001??1111111110?1?1?00111012111111?1102?1111??012101000000100101000000010??????????100001100011110011?11111010211012102001211011?0111211?11?0111200000

Echinodon_becklesii ?????0--?????0?1????0?????1?????????????????????????????????????????????????????????????????1????????00?0?00?0???0?0??10?1100100?????0????00???????????????????????????????????????????????????????????????????????????????????????????????????????????????????????????

Emausaurus_ernsti ?00010--?0??00?1?0?00?00?000001?010??10101?0?000-?0????1?0000010001?0????????????????0?10????????????1??1?0?03?00000101001100??0?????00???00???????????????????????????????????????????????01?????????????????????????????????????????????????????????????????????0011?

Eocursor_parvus 0???????????????????????????????????????????????????????????????????????????1??????????0???0?????????10?0?000?00000???10011?01?0?????0????00???0?0?????1???????????????000000????0?000?0????0?1?10??1?000000?0110000110000110000000000?200?000?11?0????????????????0000

Fruitadens_haagarorum ?????0--???1?0?1???????1?01??????????????????????????????????????????????????????????????????????????0??0?0??0?????0101001100100?????11????0?????0?1???2?????????????????????????0?010?0???????1??????????????????????????????????0011?201?000?10?1?012????????????????

Gasparinisaura_cincosaltensis 00?00???????????0?????????10111?0??01100010?00?0-0020??000?000000021?0???1???000?0?????????0????????1?20010?1101001???10?111111??111?00??????????011???20111????1101100?0?010100??000010????????????10100110011111001111101100120101111200?000011001012?211011111200000

Goyocephale_lattimorei ?1?000--?00??0??001000011010???1210-?11-10?1?01110???????01?1000?0????????????10000110????0??1???????0??00001??1?10011101?1?10???????1?????0????????????????????????11??????????21??01?1????????????10?1111??012???????????????????????????????1?????????1?000????00000

Haya_griva ?0?000--0001001?00010010?01001110?11?10011011000-?010??0000000100011000??1??00001001?0?10001121?0?101110000?12010000101001100?10?0?0?00?0??00???10?0110202?10?00101000000000001010?000101??0000?????10?001100?1200001111101100?20?011112000000?11?0??1???1???0??0000000

Heterodontosaurus_tucki 010000--01?11011000000010010001101001100010?0000-00200?0010000100001000201001010?00?10000000121?00?11000000011000010010011111011?001?11000010110001110?2???0000010000000010101?0?0?0101020010011100110000110001100001100001100000?00110201?000?10?1?0???010??0110100000

Hexinlusaurus_multidens 010000--????0????0????????100111??0001000101?000-0001??000010001001001???????01??0?????????????????????00000130??00???1001101000?1?2??01??0000??101011?1?01?0??01??0000100010??0?0?00000?0?000000000100000100011100011111011000?01000002001000?11?0?011??1???011?100000

Homalocephale_calathocercos ??1000--??????????????????10??2?2?0??11-10021011100000?000111000001?00000???101010???1???0????????????????????????0???10111????????????????????????????????????????011??????????2???????????????????10?1111??01211?011311?1111?001011??100?10??1??1001???1??????????000

Hypsilophodon_foxii 000000--11010011000000100010111?0110110001011000-00100?0000000000011100201000000100110110001121000001110000012011010101011111110101100000001210110?01102011100?11110100000010110100000001000000000001010011000121100111111110002010111020000000110000110111000110000000

Iguanodon_bernissartensis 000000--0111110101?0010?001001101101110010011000-012011?0000000100111100?00002?0001000?20001120110100131010213010011??1111111110111110011101212111?1211?21111200111012000000011020000000201001000110100001102011111011011?111112111011011002110110011110?01100111200000

Isaberrysaura_mollensis ?0000????0??00???0????00?01000?1??0??11010?010?0-???01?110000000001?????????????????????????11?????0???????????????0101???1001?????????????0?????????????????????????0??????????????????????????????100?0???????00??????????????????????????????????????????????????0??

Jeholosaurus_shangyuanensis ?00000--10011011?00000101?1001110?1?110001011000-0010??001100010001110?000????00?0?1???10??1121100001110020?1?0100001010?11??110?0?0?00101?0????00101?020?110???1??000??100?00???00??010????????????100001100011110011???011000200011102000000011?00011??1???0??0100000

Koreanosaurus_boseongensis ?????????????????????????????????????????????????????????????????????????????????????????????????????????????????????????????????????????????????0210?1?0??????00????-?000111100100000011????????????????11?0?2??1???????????01???01111200?00101110?????????????????000

Kulindadromeus_zabaikalicus ??????--????1????????????000111?0200110101001000-??01????0000000001100??????????????????????1?????????0?000003010000??10?11101?0?????00100000?????????1????00???0???0-?100010??????0001101??????????10000100001200001111?0?1?0120?01???200?0??0?1??????????0????0000000

Lesothosaurus_diagnosticus 000000--0001001100?00?00000000110000110001010000-00000?000000010001000010100000000?1?0?10000100000000100000003000000101011100100001000000000?00000?1?1???0000???1???10?000010100?00000000000000000?0100000000001000011000011000101000002000000011?00?1?0?0????110100000

Laquintasaura_venezuelae ???????????1??????????????0?0?1???1???????????0????????????00????0????01??????00?????0?????????????????????????????00?1?0?1011?0???????????0???????????????????????0????000?0???????????????????????1???0000??0?00?011?00??1?0?101?00??200??01?11????0????????????0????

Liaoceratops_yanzigouensis ?0110101?0?11010?00010001?110011?10?1??????01111010101?00012010001100002?1??0001?001????001?120?0100?10?00121??111001010111110?0?????00????1???????????????????????????????????????????????????????????????????????????????????????????????????????????????????????0???

Lycorhinus_angustidens ??????????????????????01??100?1??????????????????????????????????????????????????????????????????????????????1???????110111?01?1??????????0????????????????????????????????????????????????????????????????????????????????????????????????????????????????????????????

Macrogryphosaurus_gondwanicus ????????????????????????????????????????????????????????????????????????????????????????????????????????????????????????????????????????????????00?121?2??1???111??1????????????????????????????????1??0????0?1?11001101?01100??01?????????????????????????????????????

Mochlodon_suessi ????????????????????????????????????????????????????????????????????????????????????????????1???????11120101101??0????1???1?11??1012?00?00112?0?????????????????????????????0?????????????????????????????????????????????????????0????1??0????????????????????????0???

Mochlodon_vorosi ??????????????????????????????????????????????????????????????????????02????????????????????1???????110201011011??????10111111?01012?00?00111?????????????????????????????010101??11001?21????????????????????????????????????????0011?1000??1?11??????????????????????

Morrosaurus_antarcticus ???????????????????????????????????????????????????????????????????????????????????????????????????????????????????????????????????????????????????????????????????????????????????????????????????????????????????????????????????1110????111???1??????2??110?????????

Muttaburrasaurus_langdoni ??0?????????0????100??????1??11?1?011??????0?0????120??0100?0?01??1101???????00?00?????????????????????????????1000?????1?111110?011????00?1????111?1??2?0??????????????00010?1???00000?1?00?????1????11?01012201?00111??????0????00111200?111111?0101?02??1????01??000

Orodromeus_makelai 00?000--1001001100000?100110001?0?1?110?01011000-?01000011000000001000?210?100001??111?10??01????0?01111000?1?010000101011100100000?000100000000102111030211000?11000000001101001?00001?10000?00000010100110001211001111101100020101?1120000?[0 1]01?1001?10110?0?110000000

Nanosaurus_agilis ????????????????0???????????????????????????????????????????????????????????????????????????????????0?1???00?????0????10??11010?00120???00?00000?010?10200110??111?0?0000000010010?0000?0?0??0??????10?00?000011110011111011000201011102000000011?00011?111000110100000

Parksosaurus_warreni 00?00???????0????????0?0??10111001??110?0?0??00???010??0?0?00000?02?001????0?????????0?????1????????1???000?130??01???101111?1001012100100001001???1?10??011??01110110?00001010010???00?1???????????10100?1000?????011111?11?0110101111210?????110000?????0??0110000000

Psittacosauridae 11110100?0?11010?0?11?001011--11??0??10102?11[0 1]10-?0101?001?0001010100000????000110??1010001?110?0000?1[0 1]?0?11110[0 1]0001??10111100?0???0?00???010???10??[0 1]0???1?????01??010100[0 1]010??010?000???0?00?00000010?00110???21[0 1]?011210?112010010[0 1]???200??00?11?0???1??0????11?000000

Rhabdodon_priscus ????????????????????????????????????????????????????????????????????????????????????????????????????1?1101?1100??0????101???1??01????001001?1?????????1????1?????0?????????????????????????????????????????????????????????????????1?1??0?01011???????????????????00000

Rhabdodon_sp1 ????????????????????????????????????????????????????????????????????????????????????????????????????1?21010??0??00????101??111??10?20001001?1?????????1????1?????0??????00010200?????????????????????????????????1?01101?????1120?11?1?100010111?0?????????????11?00000

Scelidosaurus_harrisonii 000010--???100????0????0?00000102?0-?11?20?0?000-?01?0?100010010001?0?01????00?0?????0?111???????????1??1?0?030100001010111001?0?????00???000???00??01???0??0??0???0?00000?????0?0?000?0?0??????????11010000?000001111000?110??0010000?210?000?11?0??1???0????11?011111

Scutellosaurus_lawleri 0??010--0?0??0110?000??????0??????1??????????0?????????11000001000?????1????????????????????????????0?00000?03???0?0?010011001000000??0000000?00?01111?1??????0???????????010100?0?000000?000?0??0?01??00000?0?1??00110??01100000000000200?000?11?0?????????????0?00110

Stegosauria 000000--?0?10011?0?00?[0 1]00010?010220??11?20?01000-001?????0000000001?0011????00[0 1]0????10011101110000001100100?0300000[0 1]--10011001000????00????0????10?1[0 1 2][0 1 2]??????1??????0[0 1]00010010????0??00?0???01?0?001011?10000?000001[0 1]11010?1100100110???01???00?11?010101???000111210110

Stenopelix_valdensis ???????????????????????????????????????????????????????????????????????????????????????????????????????????????????????????????????????????????????????????????????000??1???????????????????????????1001111??01110?01121??1110?001011??????????11?0???????????11?00?000

Talenkauen_santacrucensis 0???00--?00101?1?0000?00?010??1?????????????????????????0???????????????????????????????????121?0?10?11000011????0?0??10?11?111???11?0?1????1???00?111??????????1????00000100??0?000?100????????????10?00111?1111?0011011?11???????111?2?0?????1??011121???110?1?000000

Tenontosaurus_dossi 00?000--010?110101000000?01011100101110001111000-0110??0?0?00000101100?00?00?21000??011??000120110001120010?110?0000??1?11111110?0??10011001?12101??2??21??11??011?010000000010010?0001010000?00???010100111?111110011011?11001201?1110210??11?1100101??1?0???110000000

Tenontosaurus_tilletti 000000--?101110101?00100001011100101110101111000-01100?000000000101100?000000210000101110000120110001120010111010001??1111111110101210011001212101?12112111111?01100100000000100100000101000000001101010011101111100110111111012011111021001110110010111110100110000000

Thescelosaurus_neglectus 000000--10010011?0000010001011101101110110011000-0010?00000000001011001000110110000100110??1121?00101120000113010000101011101110?01200010001?0101011110201111?11??101000000000000000000000000000000010100111?1111000111110110?020101?11210?1?0?11?0?0110?1???0110000000

Thescelosaurus_assiniboiensis ????????????????????????????????1??1?101???1??00-0???????00?????????????0?10?1??0??1?1?1??????????????????????????????????????????????????????????????0???11??????????????????????????????????????????100??0?1????0??11?????????????111?1?011?11????0?1????0101100?????

Tianyulong_confuciusi 00???0--?1?110???0000001??10?01?????1?????0????????????0?0?????????????2???????????????????111??00??????0?0?1000?000011002??010??????10???0?????????????????????????10????0?0??0?????0?0??????????????1?????????????1?????111??00?0?????????00?1?????????????????100000

Vegagete_ornithopod ??????????0??0???????????????????????????????????????????????????????????????????????????????????????????1?0??1??0?0??101?11111?1012000?0001201??0?1?????????????1?????????10?0???110?1?2??????????????????0???????????????????????0111???0101111?0101?????101111100000

Wannanosaurus_yansiensis ???00????????????????????????????????11??????01110????0?101?10??00??????????????????????????1?1??????0??00001??1000???10011?00?????????????0?????020?????????????????????????????1??01?1????????????1??1111???????????????????????0?????00?????10?1????????????????0???

Yandusaurus_hongheensis ???00????????????????????0100?1?01????????????????0?0???00000000?0???01?????????????????????????????0?????????????????101111101??010????0????0???0????0????1?????1?????0000??100???000000?0????????????????????????????????????2???????????000???0??????1???????0?0????

Yinlong_downsi 1111010100?1?010001000001?101011000011000200?1110?01?1?000100110111101?201??00?1100111?20??0101?0010?10?0?001?0011001110?11?00???0-0?00????????????0????????0???11?00??00?010??????????????????????010011110?011?000112100111?10??0??1?20??0??????0?0????01??????000000

Yueosaurus_tiantaiensis ?????????????????????????????????????????????????????????????????????????????????????????????????????????????????????????????????????????????????021??1????10???01?00-?000?10????????0?0????????????????????????????1?????110?????0??112001????1?101??????????????0?000

Zalmoxes_robustus 00?000--100100010001000??0101?1?1?01???????01000-10?00?01000?00110100102120??0?0010001?10??1120000101112010110010001??10111111101012000100111??11110??131??11????1?0????00010?0???11001121??????????1011011022211?1???????????1011111111100101111?01?1?????????????0000

Zalmoxes_shqiperorum 0??00?????????????????????????????011???????1000-1????00100???01101?0?02120??????100?1?10???120000??111201011001?00???10111111?0?????001001110?1???0???3??1??????1??1???11010101??1100112???????????101101102221111?111110????1011111111100101?11?0??1?????????????0000

Zephyrosaurus_schaffi ???000--1001?01110000??0?110??1?0?11?101010??0?0-?0?0000110000?0?0???0?001010010?00111110???????????1??????????????010101111?000101200??00?01000??21??0??2?1?00??1????????1??10?????????0????????????????????????????????????????????11??????????1??0??01?0?????0??????

;

proc /;

comments 0

;
