# Supplementary material for: A new basal ornithopod dinosaur from the Lower Cretaceous of China
Source: PeerJ. 2020 Sep 8;8:e9832. doi: 10.7717/peerj.9832 (PMC7485509; doi:10.7717/peerj.9832)
Supplement: Supplemental Information 4 — Character transformations were evaluated under unambiguous, fast and slow optimisation options in Winclada [40]; unambiguous synapomorphies are those that diagnose a node under both fast and slow optimisations. The synapomorphies supporting each clade are indicated by a pair of numbers. The number to the left of the dash is the character number corresponding in the list in Appendix 2, whereas the number between brackets represents the character state. Numbers in bold are unambiguous and unequivocal (CI = 1) synapomorphies. [file peerj-08-9832-s004.docx]

**Table S3. Character support for selected node, resulting of the analysis of the present dataset.**

Character transformations were evaluated under unambiguous, fast and slow optimisation options in Winclada [40]; unambiguous synapomorphies are those that diagnose a node under both fast and slow optimisations. The synapomorphies supporting each clade are indicated by a pair of numbers. The number to the left of the dash is the character number corresponding in the list in Appendix 2, whereas the number between brackets represents the character state. Numbers in bold are unambiguous and unequivocal (CI = 1) synapomorphies.

| **Node** | **unambiguous** | **ACCTRAN** | **DELTRAN** |
| --- | --- | --- | --- |
| Heterodontosauridae | 10(1), 24(1), 27(1), 52(2), 94(2), 100(1), 109(1), **135(1)**, 202(1), 229(1), 230(1), 243(1) | 2(1), 72(2), 79(1), 95(1), 101(1), 132(1), 142(1), 143(1), 150(0), 183(1), 203(1), 243(1), 247(2), 250(1) | 27(1), 207(1), **234(1)** |
| Genasauria | 27(1), **45(1)**, 63(0), 92(1), 94(1), 101(1), 145(1) | **37(0)**, 41(1), 42(0), 81(1), **112(1)**, 121(1), **155(1)**, 216(1) | 193(0) |
| Neornithischia | 95(1), 109(1), 126(0), **168(1)**, **209(1)**, **224(2)**, **247(1)** | 40(1), 56(0), 79(1), 81(1), **87(1)**, 118(1), 127(1), 148(0), 165(0) | 112(1), 131(1), **155(1)**, **162(1)**, 216(1) |
| *Hexinlusaurus* + Cerapoda | **30(1), 136(1)**, 203(1), 207(1), **215(1),** **217(1)**, 250(1) | **2(1),** 41(0), 42(0), **72(0)**, **99(1**), 203(1) | 148(0), 207(1), 226(1) |
| Cerapoda | 202(1), 208(2), **228(1)**, 258(0) | 13 (1), 37(1), 79(0), 124(1), 147(2), 152(3), 154(1), 183(1), 208(2), 229(1), 230(1), 231(1) | 40(1), 45(1), **72(0)**, 81(1), **87(1)**, 121(1) |
| *Kulindadromeus* + Marginocephalia | 184(1), 223(1) | **1(1)**, 3(1), 16(0), 19(1), 25(1), 29(1), 88(0), 92(0), 150(0), 151(1), **167(1)**, 221(1) | 13(1), 124(1) |
| *Marginocephalia* | **47(1)** , 59(1), 224(0) | 30(0), 42(0), **48(1)**, 110(1), 165(1), 168(0), 210(1), **215(2)** | 2(1), 3(1), 25(1), 221(1), 229(1) |
| Ornithopoda | **9(1)**, **23(1)**, 35(1), 52(1), **185(1)** | 2(0), 110(2),**154(2)**, 171(1) | 152(3), **231(1)** |
| Ornithopoda more derived than *Changmiania* | **36(1)**, **103(1)**, **156(1)**, 168(0), 210(1) | 13(0), 94(2), 118(0), 126(1), 199(1) | 229(1), 230(1), 249(1) |
| Orodrominae | **26(1)**, 57(1), 58(1), 76(1), 86(1), 148(1), 242(1) | 30(0), 92(0), 104(1), 127(0), 132(2), 238(1), 251(0) | 147(2), 154(2), 171(1) |
| Jeholosaurinae | 63(1), 174(0) | 96(1), 108(1), 124(0), 162(0), 199(0), 226(0) | 110(2), 183(1) |
| *Nanosaurus* + *Yandusaurus* + Clypeodonta | 160(1) | 32(0), 71(1), 125(1), 154(0), 165(1), 183(0) | 124(1), 208(1), 251(1) |
| Clypeodonta | 29(1), 129(1), 132(1), 141(1), 154(1) | 140(1), **144(1)**, 148(1), 249(2), **253(1)** | 32(0), 125(1), 165(1), 199(1) |
| Parksosaurinae | 36(0), 115(1), 172(1) | 10(1), 72(2), 99(0) | 144(1) |
| *Thescelosaurus* + Iguanodontia | 35(0), 65(1), 74(0), 81(0), **85(0)**, 108(1), 157(1), 236(1), **237(1), 239(1)** | 33(1), **78(1)**, 79(1), 86(1), 143(1), **158(1), 159(1)**, 206(1), 244(1) | 99(1), 140(1), 148(1) |
| Iguanodontia | 15(0), 23(0), 151(1), **153(1)**, 1**94(1),** 223(1), 238(1), **252(1)** | **51(1)**, 52(2), 64(1), 70(1), 71(0), 95(0), 106 (1), 175(1), 195(1), 247(2) | 144(1), 244(1), 249(2) |
